# EartEarthworm hydrolysate alleviates *Escherichia coli*-induced enteritis by suppressing MAPK pathway activation, enhancing intestinal barrier integrity, and modulating gut microbiota

**DOI:** 10.3389/fmicb.2026.1810555

**Published:** 2026-05-04

**Authors:** Jungang Kang, Sijia Jiang, Yang Li, Jinyan Zuo, Qing Yang, Weifeng Zhu, Guojun Jiang

**Affiliations:** 1 College of Veterinary Medicine, Hebei Agricultural University, Baoding, China; 2 Institute of Chinese Materia Medica, China Academy of Chinese Medical Sciences, Beijing, China

**Keywords:** earthworm hydrolysate, *Escherichia coli*, gut microbiota, intestinal inflammation, MAPK pathway

## Abstract

*Escherichia coli* enteritis causes substantial economic losses in animal husbandry, while antibiotic treatments face challenges of resistance and residue. This study investigated earthworm hydrolysate (EH) as a potential antibiotic alternative. *In vitro* assays demonstrated potent antibacterial activity of EH against *E. coli*, with an inhibition zone diameter of 12.24 ± 0.45 mm, a minimum inhibitory concentration (MIC) of 375 μg/mL, and a minimum bactericidal concentration (MBC) of 750 μg/mL. An *in vivo* enteritis model was established via intraperitoneal injection of *E. coli* (1 × 10^7^ CFU/mL). Three-day EH treatment effectively alleviated intestinal injury, improved mucosal architecture (increased villus height/crypt depth ratio), and upregulated tight junction protein (ZO-1, Occludin, Claudin-1) expression. Mechanistically, EH downregulated the levels and expression of pro-inflammatory cytokines (TNF-α, IL-6, IL-2) and inhibited the activation of the MAPK signaling pathway (reduced p-p38, p-JNK, and p-ERK). Furthermore, EH modulated gut microbiota composition, increasing the Firmicutes/Bacteroidetes ratio and beneficial genera (Bacteroides, Lactobacillus), while reducing Proteobacteria and pathogenic genera (Helicobacter, Desulfovibrio). In conclusion, EH exerts therapeutic effects against *E. coli* enteritis through multi-target mechanisms involving antibacterial action, MAPK-mediated anti-inflammatory activity, intestinal barrier restoration, and microbiota modulation, offering a promising antibiotics alternative for managing intestinal infections.

## Introduction

*Escherichia coli* enteritis is an inflammatory intestinal disease caused by pathogenic *E. coli*, representing the most common form of bacterial enteritis. Clinically, it manifests as symptoms such as diarrhea, abdominal pain, and fever, leading to high mortality rates, growth retardation, and significant economic losses (Zhang et al., 2024). Antibiotics are the primary treatment, however, they readily induce bacterial resistance, leave drug residues, and exacerbate environmental pollution. Consequently, many countries have enacted legislation prohibiting antibiotic additives in animal feed (Liu et al., 2025). While corticosteroids and immunosuppressants are also used to treat enteritis, their clinical efficacy is often suboptimal and accompanied by significant side effects. Therefore, there is an urgent need to develop a highly effective, residue-free antibiotic alternative.

Research indicates that animal protein hydrolysates possess multiple bioactive properties, exhibiting antioxidant, anti-inflammatory, and antibacterial effects. For instance, sheep plasma protein hydrolysate extends the shelf life of meat products through its anti-inflammatory, antioxidant, and antibacterial actions (Kumari et al., 2024). Fish protein hydrolysates also demonstrate similar efficacy (Santos-Sánchez et al., 2021; Shekoohi et al., 2024). EH contains abundant protein hydrolysates with unique antibacterial and anti-inflammatory effects due to its diverse antimicrobial substances and enzymes (Sung et al., 2024). Compared to other animal proteins, EH offers advantages in safety, cost, bioavailability, and antimicrobial efficacy, making it a promising green remedy against *E. coli* enteritis. Earthworm antimicrobial peptides (EAP), small-molecule polypeptides produced by the earthworm immune system, play a crucial role in innate immune defense by disrupting microbial cell membrane structures and functional groups (Venkatachalam et al., 2025; Wani et al., 2025). This study compared them with the crude hydrolysate to investigate whether they are the primary antimicrobial components.

The animal gut harbors a complex microbial community, whose composition and function are closely linked to host health. Proteolytic products and bioactive peptides are recognized as key modulators of the gut microbiota, capable of improving metabolic health by altering microbial composition (Aloo and Oh, 2022). A bidirectional relationship exists between intestinal inflammation and gut microbiota: inflammation often accompanies dysbiosis, and conversely, dysbiosis can trigger inflammation, which can be exacerbated by abnormal microbial metabolites (Dong and Moon, 2025). Therefore, we collected cecal contents from the region with the highest density and activity of gut microbes for 16S rRNA sequencing to investigate the effects of EH on the intestinal microbiota.

In summary, systematic studies are still limited on the comprehensive regulatory effects of EH on bacterial enteritis, particularly regarding its impact on intestinal barrier function, signaling pathways, and the microbiota. This study employs *in vitro* antibacterial assays and an *in vivo E. coli* enteritis model to comprehensively evaluate the therapeutic efficacy of EH and to investigate its mechanisms of action, including the regulation of the MAPK pathway, inflammatory response, tight junctions, and gut microbiota. This not only provides evidence for the therapeutic potential of EH in treating intestinal inflammation but also offers theoretical guidance for developing novel antibiotic alternatives for bacterial enteritis in animals.

## Materials and methods

### Animals and reagents

Eight-week-old SPF-grade Kunming mice, with equal numbers of males and females, were purchased from Spefo (Beijing) Co., Ltd. All mice were housed under specific pathogen-free conditions with a controlled environment: room temperature was maintained at 22 ± 2°C, relative humidity at 50% ± 10%, and a 12-hour light/dark cycle. Mice were housed in polypropylene cages with sterilized corncob bedding, and standard laboratory chow and autoclaved water were provided *ad libitum*. After one week of acclimatization, subsequent experiments were conducted. *Escherichia coli* (1919D3) was provided by the College of Animal Medicine, Hebei Agricultural University. EAP (purity > 98% as confirmed by HPLC and mass spectrometry) were purchased from Shanghai Jier Biochemical Co., Ltd., and Gentamicin sulfate (GM) injection (2 mL: 80,000 IU) was manufactured by Shanghai Quanyu Biotechnology Animal Pharmaceutical Co., Ltd. For use in experiments, EAP was dissolved in sterile PBS to a working concentration of 1000 μg/mL, and GM was diluted with PBS to a working concentration of 512 μg/mL. Both solutions were stored at 4°C. All experimental animal use and procedures in this study were approved by the Animal Ethics Committee of Hebei Agricultural University (1351651651).

### Bacterial strain recovery and activation and culture

The *E. coli* strain was retrieved from the −80°C freezer. After thawing, 1 mL of the bacterial stock was inoculated into 3 mL of Luria-Bertani (LB) liquid medium and incubated at 37°C with shaking at 180 rpm for 12 h. This initial culture constituted the first activation passage. To ensure the bacteria were in the mid-logarithmic growth phase and to restore optimal physiological activity prior to experimentation, the activation process was repeated twice for a total of three successive passages. Following the third passage, the bacterial culture was harvested at the mid-log phase (optical density at 600 nm, OD_600_ = 0.6–0.8) to ensure uniform and reproducible metabolic activity for subsequent experiments. No additional inducing agents were used during activation, as the aim was to maintain the strain in its standard planktonic state for consistent *in vitro* and *in vivo* challenge. To determine the bacterial concentration, the purified *E. coli* suspension was subjected to 10-fold serial dilutions, yielding nine concentration gradients ranging from 10^–1^ to 10^–9^. From each dilution, 100 μL was spread onto LB agar plates, with three replicate plates per dilution. The plates were incubated upside down at 37°C for 24 h to obtain countable colonies. The concentration of the purified *E. coli* suspension was determined to be 1 × 10^9^ CFU/mL by colony counting, and this stock was stored at 4°C for future use.

### Preparation of earthworm hydrolysate

The earthworm hydrolysate was prepared using a modified autolysis method based on a previously described procedure (Liu H. et al., 2023). Briefly, 100 g of live earthworms (*Eisenia fetida*) were washed with sterile water, blotted dry, and placed in a sterile conical flask. The flask was incubated in a 55°C water bath for 5 h to promote autolysis via endogenous digestive enzymes. The resulting yellow hydrolysate was collected and filtered through sterile gauze to remove cellular debris and particulate matter. The total protein concentration of the filtrate was determined using the BCA method to standardize the bioactive component content across different batches. Based on the measured protein concentration, the hydrolysate was diluted with PBS to a final working concentration of 750 μg/mL for *in vivo* experiments. The solution was stored at 4°C until use.

### Disk diffusion assay for antibacterial activity

A volume of 20 μL of each solution (EH, EAP, GM) was pipetted onto blank antimicrobial susceptibility discs (6.0 mm diameter). Then, 100 μL of pre-activated and diluted *E. coli* suspension (concentration 1 × 10^9^ CFU/mL) was spread evenly onto LB agar plates. After the bacterial suspension was absorbed for 10 seconds, the antibiotic discs containing the solutions were placed onto the LB agar plates. The plates were incubated at 37°C for 24 h, and the diameters of the inhibition zones were measured. The experiment was performed in triplicate, and the average diameter was calculated.

### Determination of minimum inhibitory concentration and minimum bactericidal concentration

The MIC and MBC of EH, EAP, and GM against *E. coli* were determined using the broth microdilution method. Serial two-fold dilutions of each drug were prepared in LB liquid medium within the following concentration ranges: GM, 16–512 μg/mL; EH, 23.4–750 μg/mL; EAP, 31.25–1,000 μg/mL. The bacterial suspension was added to each dilution to achieve a final concentration of 10^6^ CFU/mL. After incubation at 37°C with shaking at 180 rpm for 24 h, aliquots from wells showing no visible turbidity were spread onto LB agar plates and incubated for an additional 12 h. The MIC was defined as the lowest drug concentration that inhibited visible microbial growth, and the MBC was defined as the lowest drug concentration that resulted in no colony formation. Each experiment was performed in triplicate.

### Establishment of a mouse model of *E. coli*-induced enteritis

Forty-five eight-week-old SPF mice (equal numbers of males and females) were randomly divided into Groups (I-V, n = 9). Groups I-IV received intraperitoneal (IP) injections of *E. coli* suspensions at concentrations of 1 × 10^9^, 1 × 100^8^, 1 × 10^7^, and 1 × 100^6^ CFU/mL, respectively. Group V (control) received an IP injection of 0.2 mL physiological saline. All injections were administered at 0.2 mL. Mice were observed continuously for 3 days post-injection, and diarrhea status, general condition, and mortality were recorded daily. The diarrhea rate was calculated as the percentage of mice showing diarrhea within each group, and the mortality rate was calculated as the percentage of mice that died. Based on the cumulative observations over the 3-day period, the dose yielding the highest diarrhea rate with low mortality was selected for subsequent therapeutic experiments. To verify successful model establishment and intestinal bacterial colonization, three mice from each group were randomly selected and euthanized on day 3 post-infection. Intestinal contents (0.5 g) were collected, homogenized in saline, serially diluted (10^2^-100^4^), and plated on VBEA agar for *E. coli* enumeration using the overlay method. Plates were incubated upside down at 37°C for 24 h before colony counting. The remaining six mice in each group were euthanized and their tissue samples were collected for other exploratory analyses (data not shown), and these mice were not included in the subsequent treatment experiments.

### Animal experiment design and sample collection and analysis

Fifty eight-week-old SPF mice (equal sexes) were randomly divided five groups (*n* = 10): A (Control group), B (*E. coli* group), C (EAP group), D (EH group), and E (GM group). Based on the results of the model establishment experiment, Groups B-E were injected IP with the 1 × 10^7^ CFU/mL *E. coli* suspension to induce enteritis. Beginning at 12 h post-infection, diarrhea and mortality in Group B were observed to determine whether the model was successfully established. Twelve 12 h post-infection, based on the minimum bactericidal concentrations (MBCs) determined *in vitro* and confirmed by preliminary tolerance tests, daily oral administration was performed via gavage (0.2 mL) for three consecutive days: Group A and B received physiological saline; Group C received EAP (1,000 μg/mL); Group D received EH (750 μg/mL); Group E received GM (512 μg/mL). Body weight was measured before infection (initial) and after the treatment period (final). After the last administration, mice were fasted for 12 h with free access to water before euthanasia. The jejunum, cecum, heart, liver, spleen, lungs, and kidneys were collected and weighed for organ index calculation (organ weight/body weight × 100%).

### Morphological observations of the jejunum

The mouse jejunum segments were fixed in 4% paraformaldehyde, processed through graded ethanol and xylene, embedded in paraffin, sectioned, and stained with hematoxylin and eosin (H&E). Images were captured using a light microscope (Nikon Eclipse E100, Nikon, Japan), and villus height and crypt depth were measured using ImageJ software. The villus height-to-crypt depth ratio (V/C ratio) was subsequently calculated.

### ELISA analysis

Levels of IL-2, IL-6, and TNF-α in jejunal homogenates were quantified using commercial ELISA kits (mlsw-E3056, mlsw-E3082, mlsw-E3071, Mlbio, Shanghai) according to the manufacturer’s instructions. Absorbance was read at 450 nm using a Thermo Scientific microplate reader, and cytokine concentrations were determined from the standard curve.

### RT-qPCR analysis

Primers required for the experiment were designed using Primer BLAST software based on the gene CDS sequences in GenBank (Table 1). RNA was extracted from jejunal tissue using the Trizol method, reverse transcribed into cDNA, and processed according to the RT-qPCR kit instructions (TianGen Biochemical Technology Co., Ltd.). Relative gene expression was normalized to GAPDH as an internal control. mRNA expression was calculated using the 2^(^ΔΔ^CT) method.

**TABLE 1:** Target gene primer sequences, amplified fragment lengths, and sequence numbers.

Target gene	Primer	
	Direction	Sequence (5′–3′)
TNF-α	F	CGCTCTTCTGTCTACTGAACTTCGG
	R	GTGGTTTGTGAGTGTGAGGGTCTG
IL-6	F	CTTCTTGGGACTGATGCTGGTGAC
	R	TCTGTTGGGAGTGGTATCCTCTGTG
IL-2	F	GACCTCTGCGGCATGTTCTGG
	R	GTCCACCACAGTTGCTGACTCATC
Occludin	F	TGGCTATGGAGGCGGCTATGG
	R	ACTAAGGAAGCGATGAAGCAGAAGG
Claudin-1	F	GCTGGGTTTCATCCTGGCTTCTC
	R	CCTGAGCGGTCACGATGTTGTC
ZO-1	F	TGCTAATGCCTCGGAAAGAGATGAC
	R	GCTGTGGAGACTGCGTGGAATG
GAPDH	F	GGCAAATTCAACGGCACAGTCAAG
	R	TCGCTCCTGGAAGATGGTGATGG

### Western blot analysis

Mix RIPA, PMSF, protein phosphatase inhibitor, and bacteriocin in a 1,000:10:10:1 ratio on ice and set aside. Prepare a 10% tissue homogenate by mixing jejunal tissue with RIPA lysis buffer at a 1:9 ratio. Add pre-chilled steel beads to centrifuge tubes and disrupt tissue using a homogenizer. Incubate on ice for 15–30 min. Centrifuge the lysate at 4°C and 12,000 × g for 5 min, then collect the supernatant. Determine protein concentration using the BCA assay kit (Nanjing Jiancheng Biotechnology Co., Ltd.). Adjust protein sample concentrations to uniformity using RIPA lysis buffer. Mix protein samples with SDS-PAGE loading buffer at a 4:1 ratio, heat in a 100°C water bath for 10 min to denature proteins, aliquot, and stored at −80°C for later use. Transfer proteins to PVDF membranes using the Bio-Rad system. After blocking, incubate with primary antibodies: anti-β-Actin (bs-0061R), anti-ERK1/2 (db11649), anti-p-ERK1/2 (db13437), anti-JNK1/2/3 (db12713), anti-p-JNK1/2/3 (bs-17591R), anti-p38 (db15981), anti-p-p38 (db12597). After overnight incubation and washing, incubate the secondary antibody (bs-0295G-HRP) at room temperature for 2 hs. A pre-configured ECL chemiluminescent reagent was used for the color reaction with the detected proteins. The target bands were detected using a chemiluminescence imager. Finally, grayscale analysis was performed using Image J software.

### Immunofluorescence

Following dewaxing of paraffin sections, blocking was performed using BSA for 30 min. The sections were then incubated with primary antibodies against p-ERK1/2, p-JNK1, and p-p38 at 4°C overnight. After three 5-min washes with PBS, the sections were incubated with a secondary antibody at room temperature for 50 min. Nuclei were counterstained with DAPI, and the sections were mounted with an anti-fade mounting medium. Finally, images were acquired using a fluorescence microscope.

### Microbial diversity sequencing

Building upon previous methods (Liu et al., 2022), 12 h after the final drug administration, three mice were randomly selected from each group, and 5 g of cecal contents were collected. Nucleic acids were extracted using the OMEGA Soil DNA Kit (M5635-02) (Omega Bio-Tek, Norcross, GA, United States). The extracted DNA was subjected to 0.8% agarose gel electrophoresis for molecular size determination and quantified using Nanodrop. The highly variable V3-V4 region of the bacterial 16S rRNA gene, approximately 280 bp in length, was selected for sequencing. PCR products were quantified using the Quant-iT PicoGreen dsDNA Assay Kit on a Microplate reader (BioTek, FLx800), then pooled according to the required data volume per sample. Finally, paired-end sequencing of community DNA fragments was performed using the Illumina platform.

### Statistical analysis

Data are expressed as mean ± standard deviation. Statistical analysis was performed using SPSS 21.0 software. After confirming homogeneity of variance, one-way analysis of variance (ANOVA) followed by an appropriate *post-hoc* test was used for multiple comparisons. A *P*-value < 0.05 was considered statistically significant. Graphs were generated using GraphPad Prism 6.0.

## Results

### Antibacterial zone measurement results of EH

The average inhibition zone diameters for EH, EAP, and GM were 12.24 mm, 11.36 mm, and 13.86 mm, respectively. Data are presented as mean ± standard deviation from three independent replicate experiments. Compared with distilled water, the inhibition zone diameters of EH, EAP, and GM were significantly increased (*P* < 0.05), as shown in Table 2.

**TABLE 2:** Antibacterial effects of different drugs on *E. coli.*

Drug Name	Drug dosage/μ L	Antibacterial zone diameter/mm	MIC μ g/mL	MBC μ g/mL
GM	20	13.86 ± 0.15^a^	256	512
EH	20	12.24 ± 0.45^ab^	375	750
EAP	20	11.36 ± 0.12^b^	500	1,000
Distilled water	20	6.00 ± 0^c^	0	0

Different letters (a–c) indicate significant differences among different groups (*P* < 0.05).

### Minimum inhibitory concentration and minimum bactericidal concentration of EH

As shown in Table 2, the minimum inhibitory concentration (MIC) of GM against *E. coli* is 256 μg/mL. The MIC of EH against *E. coli* is 375 μg/mL; the MIC of EAP against *E. coli* is 500 μg/mL. The minimum bactericidal concentration (MBC) of GM against *E. coli* is 512 μg/mL; the MBC of EH against *E. coli* is 750 μg/mL; the MBC of EAP against *E. coli* is 1,000 μg/mL. EH, EAP, and GM all exhibit antibacterial activity against *E. coli*. Among these, EH demonstrates superior antibacterial efficacy to EAP but is less potent than GM.

### Establishment of an *E. coli*-induced intestinal inflammation model in mice

To establish a reliable enteritis model, mice were intraperitoneally (IP) injected with varying concentrations of *E. coli* (1 × 10^6^ to 1 × 10^9^ CFU/mL). The clinical outcomes are summarized in Table 3. The control group (Group V) exhibited normal feces and clean fur. In contrast, all infected groups developed watery diarrhea and soiled perianal fur, confirming successful induction of enteritis. The severity of clinical signs and pathological changes was dose-dependent. Notably, the 1 × 10^9^ CFU/mL group (Group I) resulted in 100% mortality, while the 1 × 10^7^ CFU/mL group (Group III) achieved a 100% diarrhea rate with a low mortality rate (11.11%). Necropsy of Group III revealed characteristic enteritis pathology, including intestinal hyperemia and flaccid bowel with watery contents. Furthermore, bacterial load quantification in intestinal contents (Table 3) confirmed robust colonization in all infected groups, with the 1 × 10^7^ CFU/mL dose resulting in a substantial yet non-lethal bacterial load (1.7 × 10^8^ CFU/g). Based on these findings, the 1 × 10^7^ CFU/mL dose was selected for all subsequent experiments to evaluate therapeutic interventions.

**TABLE 3:** Diarrhea and mortality in mice.

Group	Diarrhea	Death	No diarrhea	Diarrhea rate	Mortality rate	Bacterial load
I (1 × 10^9^CFU/mL)	9	9	0	100%	100%	9.0 × 10^10^ CFU/g
II (1 × 10^8^CFU/mL)	9	3	0	100%	33.3%	3.0 × 10^9^ CFU/g
III (1 × 10^7^CFU/mL)	9	1	0	100%	11.1%	1.7 × 10^8^ CFU/g
IV (1 × 10^6^CFU/mL)	4	0	5	44.5%	0	4.5 × 10^6^ CFU/g
V (Blank group)	0	0	9	0	0	1.6 × 10^4^ CFU/g

### Therapeutic effects of EH on mice body weight

**FIGURE 1:**
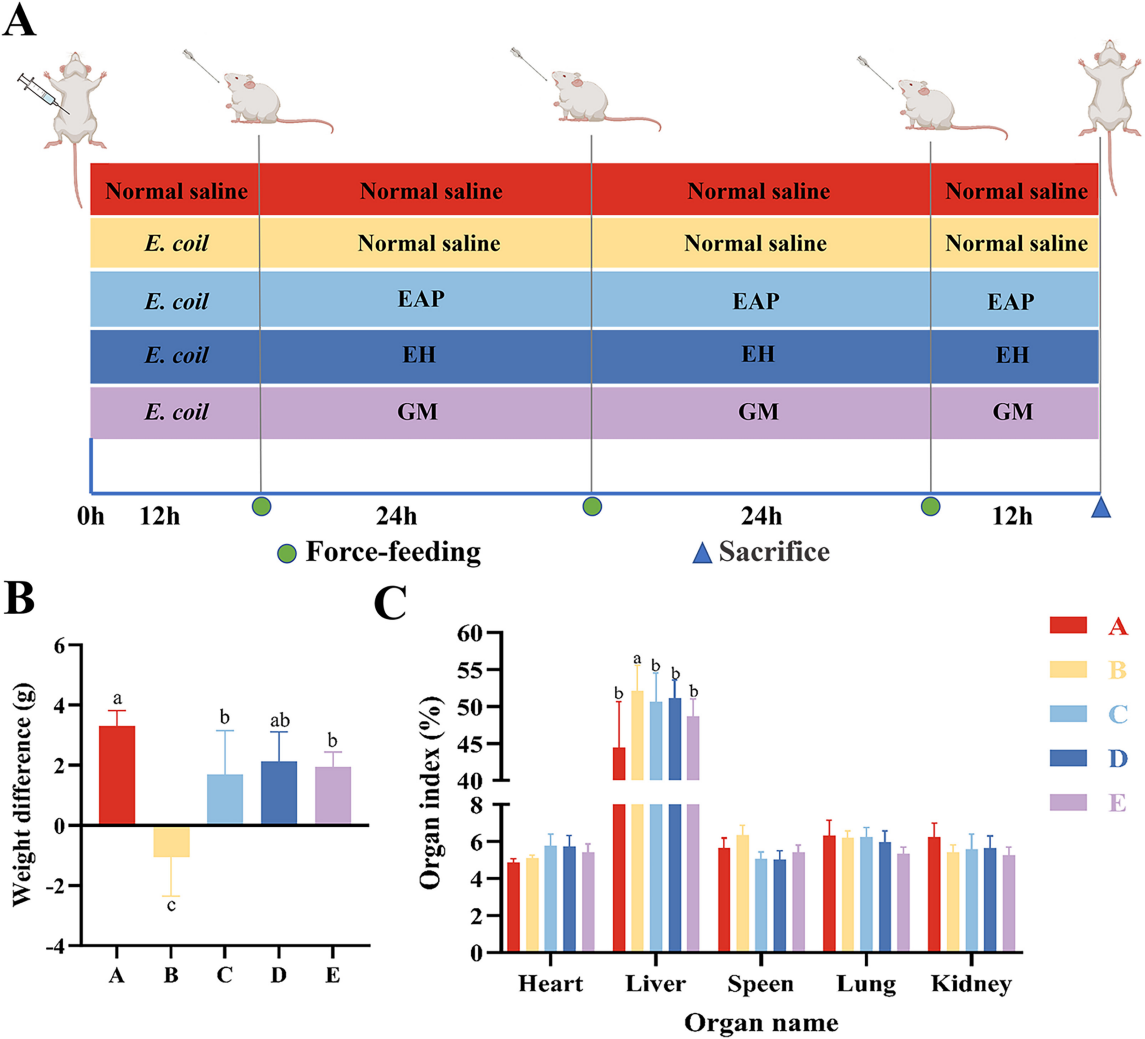
Protective effects of EH on growth and development in mice. **(A)** Schematic diagram of the experiment. **(B)** Weight difference. **(C)** Organ index. Different letters (a–c) indicate significant differences among different groups (*P* < 0.05).

The schematic diagram of the experimental design is shown in Figure 1A. During the 3-day observation period, all mice in Group B developed diarrhea, resulting in a diarrhea rate of 100%. One mouse died in this group, giving a mortality rate of 10% (1/10), which was generally consistent with the results of the preliminary model establishment experiment. Regarding the effects on weight difference (Figure 1B), compared with Group A, the weight difference was significantly reduced in Groups B, C, and E (*P* < 0.05), while no significant difference was observed in Group D (*P* > 0.05). Compared with Group B, the weight difference was significantly increased in Groups A, C, D, and E (*P* < 0.05). These results indicate that EH is superior to EAP and GM in restoring body weight.

### Therapeutic effects of EH on organ indexes in mice

Regarding liver indices, Group B exhibited significantly elevated levels compared to Group A (*P* < 0.05). Compared to Group B, Groups C, D, and E all showed significantly reduced levels (*P* < 0.05). Compared to Group E, no significant changes were observed in Groups B and C (*P* > 0.05). No significant differences were noted in other organ indices (Figure 1C).

### Effects of EH on histological changes in mice intestinal tissue

**FIGURE 2:**
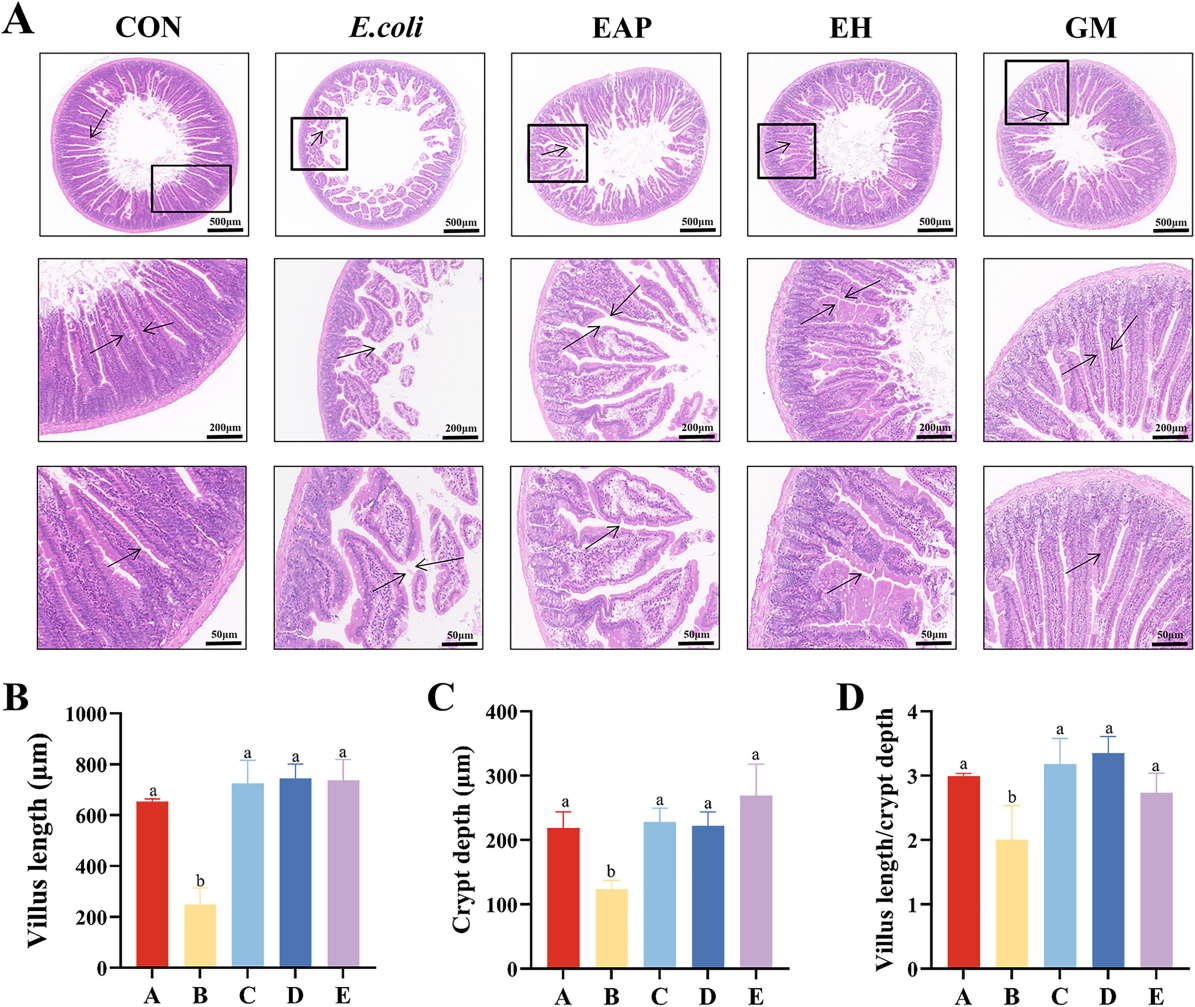
Protective effects of EH on mouse jejunum tissue **(A)** Jejunum tissue section, black arrows indicate damaged and detached intestinal villi. **(B)** Villus length. **(C)** Crypt depth. **(D)** Ratio of villus length to crypt depth. Different letters indicate significant differences among different groups (*P* < 0.05).

Histological analysis of jejunal tissue revealed that the control group exhibited intact jejunal structure with preserved intestinal endothelium and muscularis layers, and neatly arranged crypts. In contrast, the model group demonstrated epithelial cell disorganization, muscularis rupture, inflammatory cell infiltration, villus atrophy, and crypt distortion, with extensive inflammatory cell infiltration leading to disruption of villus architecture. The jejunal structures in the EAP, EH, and GM treatment groups remained relatively intact (Figure 2A). Compared with Group B, villus height and crypt depth were significantly increased in the other groups (*P* < 0.05). Calculation of the villus height-to-crypt depth ratio revealed a significantly reduced ratio in Group B (*P* < 0.05) (Figures 2B–D).

### Effects of EH on intestinal TNF-α, IL-6, and IL-2 levels in mice

**FIGURE 3:**
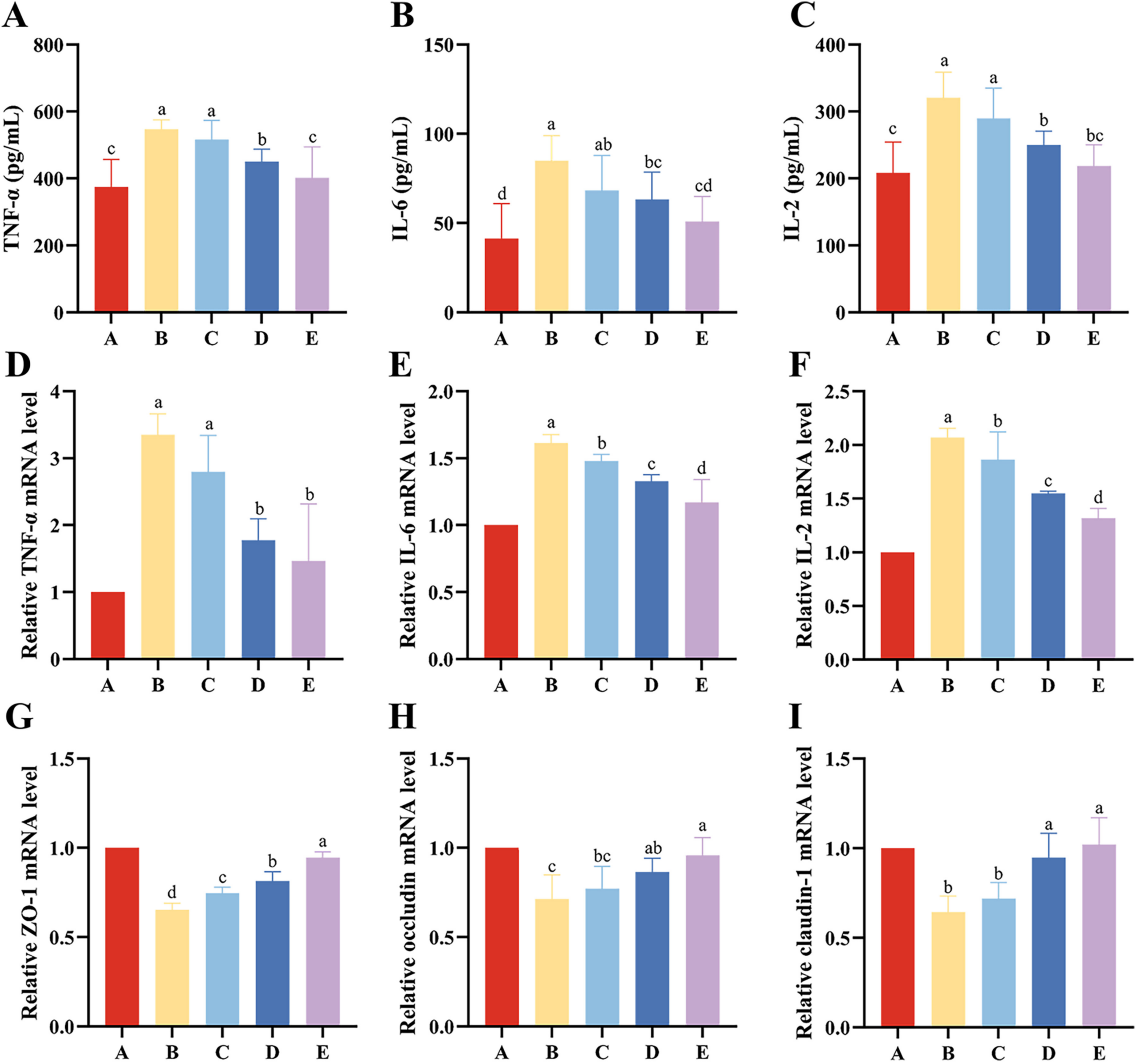
Protective Effects of EH on Inflammatory Markers and Tight Junction Proteins in Mice. **(A–C)** Inflammatory factor content. **(D–F)** Relative expression levels of inflammatory factor mRNA. **(G–I)** Relative expression levels of tight junction protein mRNA. Different letters (a–d) indicate significant differences among different groups (*P* < 0.05).

Compared with Group A, the levels of inflammatory cytokines TNF-α, IL-6, and IL-2 in the intestines of Group B mice were significantly elevated (*P* < 0.05). Compared with Group B, the levels of TNF-α, IL-2, and IL-6 were significantly reduced in both Group D and Group E (*P* < 0.05). Compared with Group C, Group D exhibited significantly reduced levels of TNF-α and IL-2 (*P* < 0.05), while Group E showed significantly reduced levels of all three cytokines (TNF-α, IL-2, and IL-6) (*P* < 0.05) (Figures 3A–C).

### Effects of EH on mRNA expression levels of intestinal inflammatory cytokines in mice

Compared with Group A, the relative mRNA expression levels of TNF-α, IL-2, and IL-6 were significantly elevated in the other groups (*P* < 0.05). Compared with Group B, the relative mRNA expression levels of IL-2 and IL-6 in Group C were significantly reduced (*P* < 0.05). The relative mRNA expression levels of TNF-α, IL-2, and IL-6 in the intestinal tissues of Group D mice were significantly reduced (*P* < 0.05). Compared with Group E, the relative mRNA expression levels of IL-2 and IL-6 in Group D were significantly increased (*P* < 0.05) (Figures 3D–F).

### Effects of EH on the expression of genes related to tight junctions in the mouse intestine

Compared with Group B, the relative mRNA levels of ZO-1, Claudin-1, and Occludin were significantly increased in Group D (*P* < 0.05). Compared with Group C, the relative levels of ZO-1 and Claudin-1 were significantly increased in Group D (*P* < 0.05), while the relative levels of ZO-1, Claudin-1, and Occludin were significantly increased in Group E (*P* < 0.05) (Figures 3G–I).

### Effects of EH on the MAPK signaling pathway in the mice intestine

**FIGURE 4:**
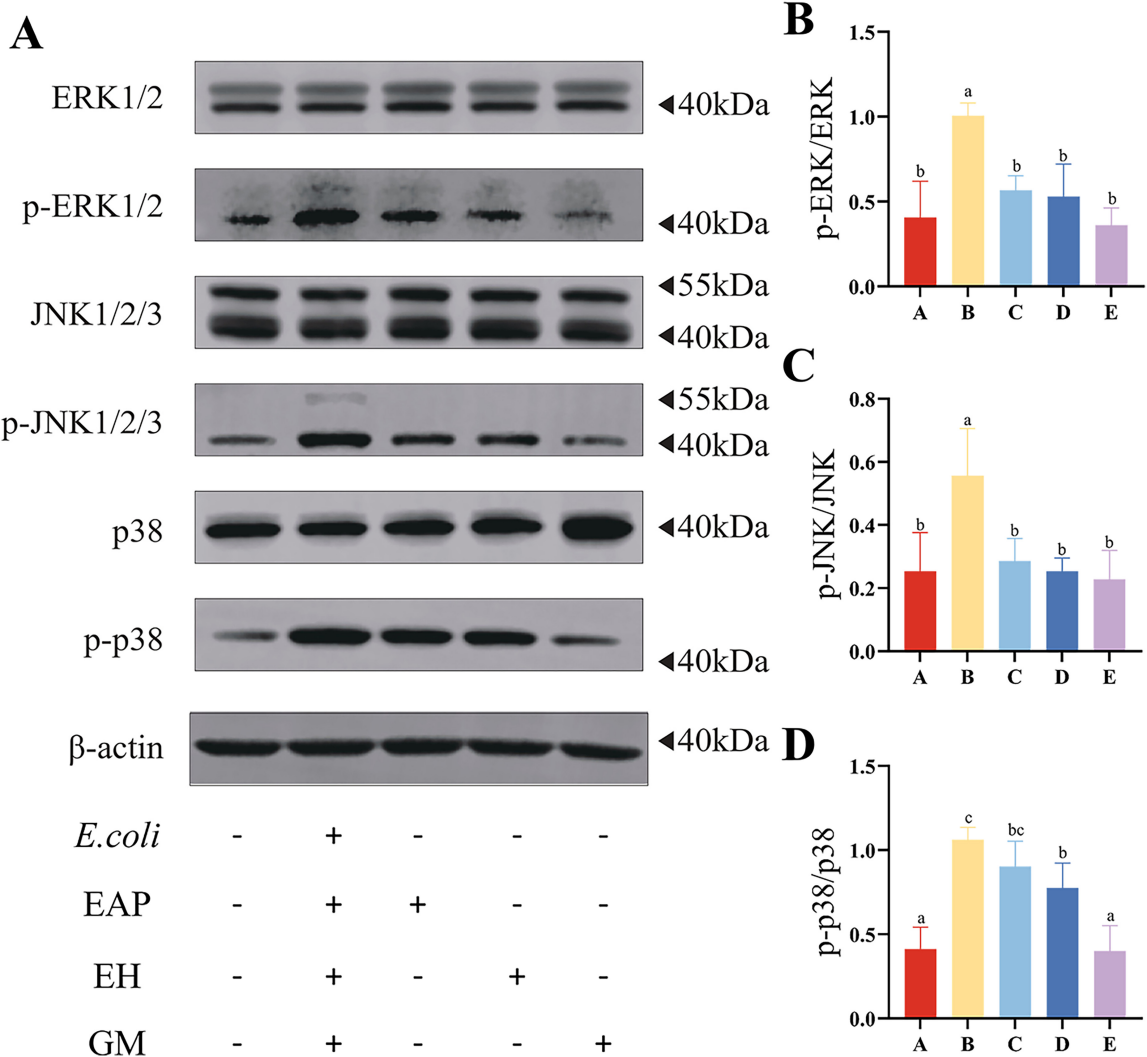
Protective effects of earthworm hydrolysate on the expression of MAPK pathway-related proteins in mice. **(A)** Protein band patterns for ERK1/2, p-ERK1/2, JNK1/2/3, p-JNK1/2/3, p38, p-p38, and β-actin. **(B)** Results of grayscale analysis of p-ERK/ERK protein bands. **(C)** Grayscale analysis results of p-JNK/JNK protein bands. **(D)** Grayscale analysis results of p-p38/p38 protein bands. Different letters (a–d) indicate significant differences among different groups (*P* < 0.05).

Compared with Group A, the ratios of p-ERK/ERK, p-JNK1/JNK and p-p38/p38 were significantly elevated in Group B (*P* < 0.05); Compared with Group B, the ratios of p-ERK/ERK, p-JNK1/JNK and p-p38/p38 were significantly decreased in Group D (*P* < 0.05) (Figure 4).

**FIGURE 5:**
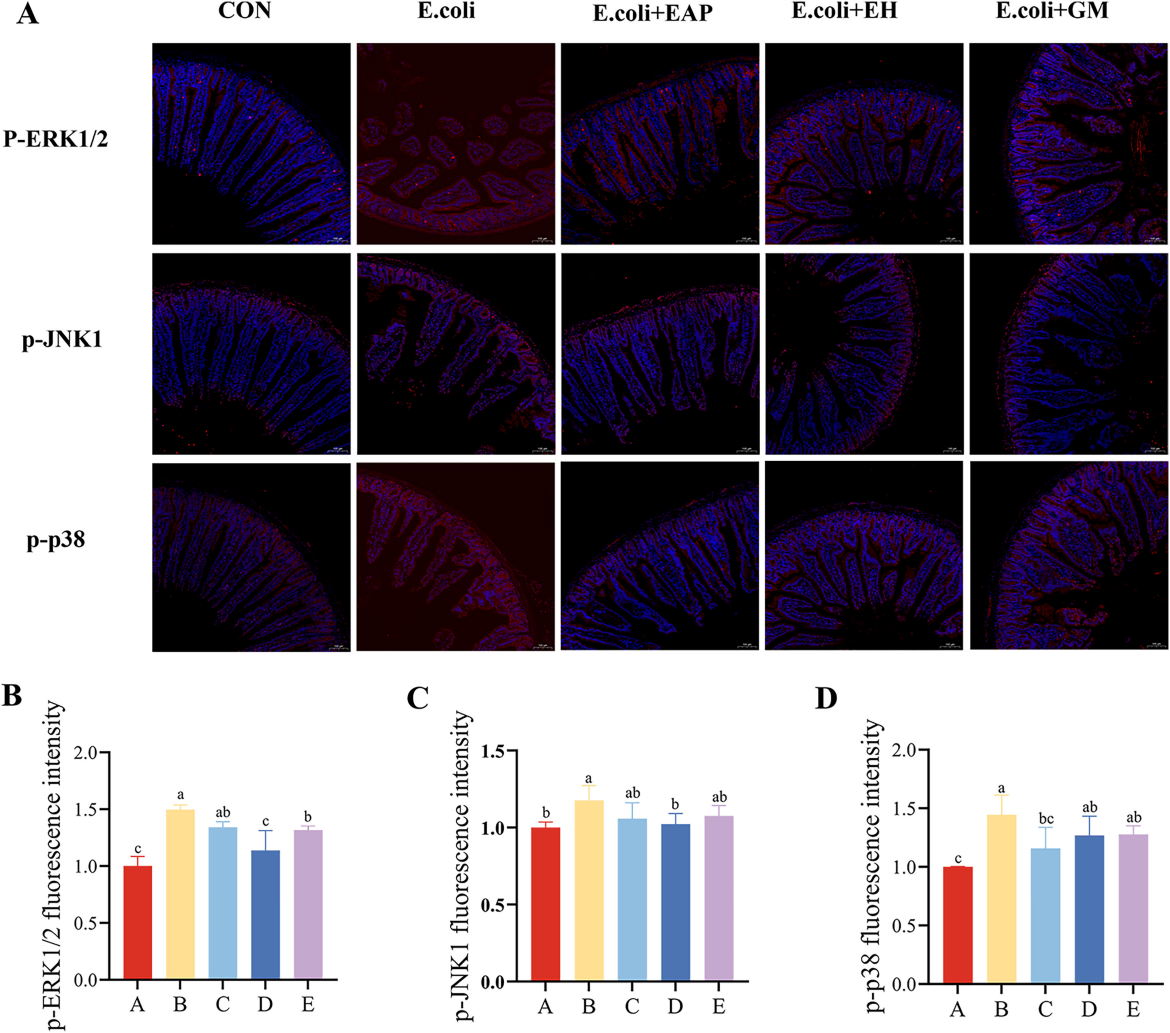
Effects of EH on fluorescence intensity of MAPK-related proteins in mouse intestines **(A)** merged fluorescent images. **(B)** p-ERK1/2 fluorescence intensity. **(C)** p-JNK1 fluorescence intensity. **(D)** p-p38 fluorescence intensity. Different letters (a–c) indicate significant differences among different groups (*P* < 0.05).

Immunofluorescence results were consistent with Western blot findings. Compared with Group A, Groups B exhibited significantly increased immunofluorescence intensities for p-ERK1/2, p-JNK1, and p-p38. Compared with Group B, C, D, and E showed varying degrees of decreased intensity (Figure 5).

### Effects of EH on the gut microbiota of mice

**FIGURE 6:**
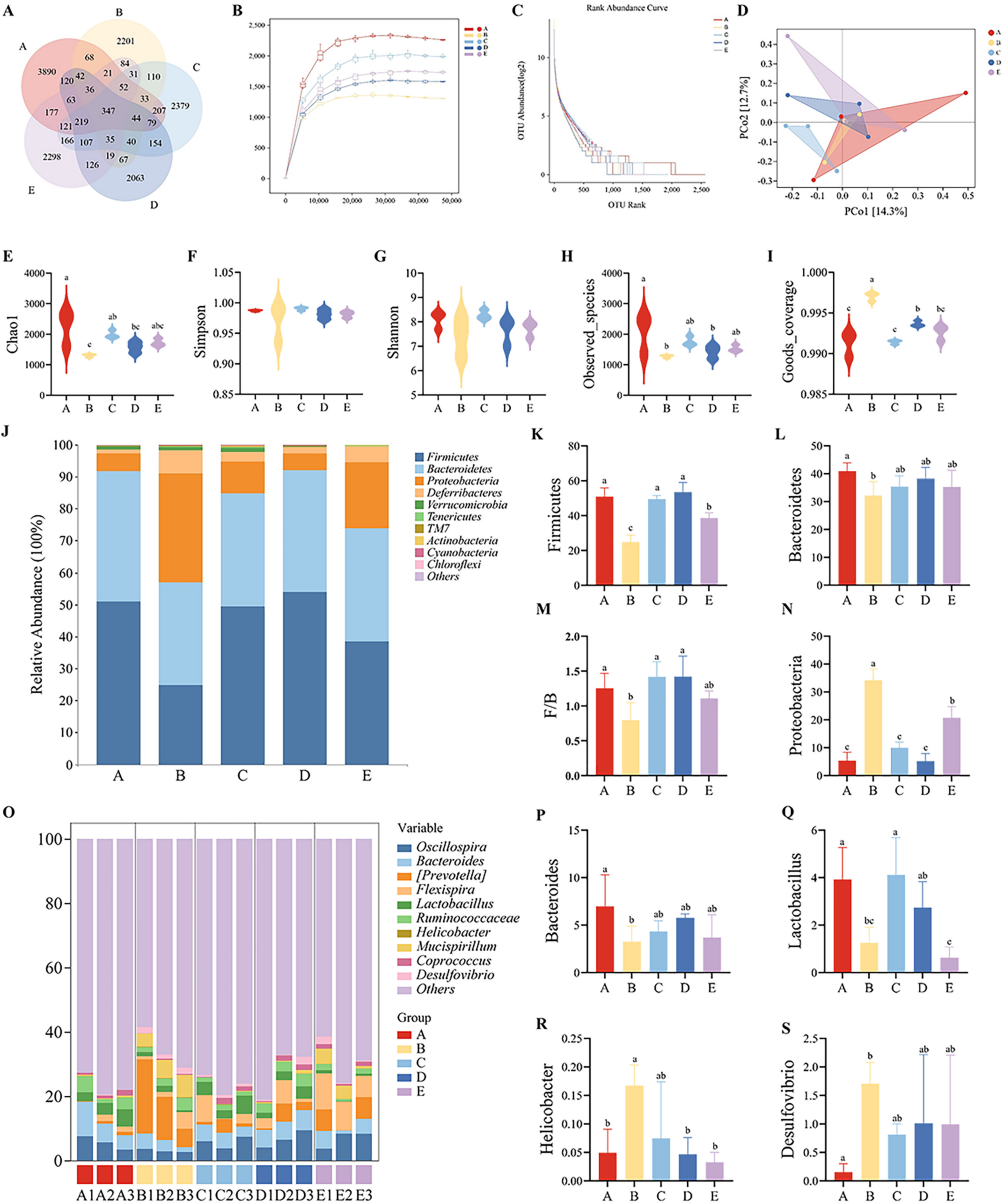
Effects of EH on gut microbiota **(A)** Venn diagram of core operational taxonomic units (OTUs). **(B)** Sparsity curve. **(C)** Abundance level curve. **(D)** PCoA analysis. **(E–I)** α diversity indices. **(J)** Relative abundance at the phylum level. **(K)** Relative abundance of Firmicutes. **(L)** Relative abundance of Bacteroidetes. **(M)** Firmicutes/Bacteroidetes ratio. **(N)** Relative abundance of Proteobacteria. **(O)** Relative abundance at the genus level. **(P)** Relative abundance of Bacteroides. **(Q)** Relative abundance of Lactobacillus. **(R)** Relative abundance of Helicobacter. **(S)** Relative abundance of Desulfovibrio. Different letters (a–c) indicate significant differences among different groups (*P* < 0.05).

This study performed 16S rRNA sequencing on cecal contents. After quality control, a total of 1,220,339 sequences were generated from 15 samples, ranging in length from 50 to 434 bp with an average length of 361 bp. Groups A-E yielded 3,890, 2,201, 2,379, 2063, and 2298 unique OTUs, respectively, with 347 OTUs shared across all groups (Figure 6A). The rarefaction curve, a common metric for describing sample diversity within groups, indicates that sequencing data volume is progressively adequate as the curve flattens. This confirms the validity of sequencing results and sufficient data volume in this study (Figure 6B). The Rank Abundance curve visually reflects species richness and evenness within a sample. The smoothness of the curve indicates species distribution uniformity—a flatter curve signifies more even distribution. Results show a broad curve with a gentle slope, indicating high species richness and evenness, suggesting good community diversity (Figure 6C). In PCoA analysis, the distance between sample points serves as a key parameter for assessing the similarity of taxonomic distributions. Points closer together in the coordinate system indicate higher similarity, while greater distance signifies greater divergence. Principal Coordinate Analysis (PCoA) revealed differences in gut microbiota structure among the four groups: the model group, EAP group, EH group, and GM group (Figure 6D). Regarding α-diversity, compared with the blank control group, both the Chao1 index and Observed_species index were significantly reduced in Group B (*P* < 0.05, Figures 6E–H). This indicates a significant decrease in both microbial richness and total species count in Group B. The Goods_coverage index exceeded 0.985 for all groups, confirming sufficient sequencing depth and high reliability of the results (Figure 6I).

To further investigate changes in the mouse gut microbiota, this study analyzed microbial composition differences at the phylum and genus levels. At the phylum level, the three predominant phyla were Firmicutes, Bacteroidetes, and Proteobacteria. Compared to Group A, Group B exhibited significantly lower relative abundances of Firmicutes and Bacteroidetes (*P* < 0.05), as well as a significantly lower Firmicutes/Bacteroidetes (F/B) ratio (*P* < 0.05). The relative abundance of the Proteobacteria phylum increased significantly (*P* < 0.05, Figures 6J–N). At the genus level, analysis of the top ten genera revealed significant differences in Bacteroides, Lactobacillus, Helicobacter, and Desulfovibrio. Compared to Group A, Group B exhibited significantly reduced relative abundances of Bacteroides and Lactobacillus, while Helicobacter and Desulfovibrio showed significant increases. Groups C, D, and E demonstrated a trend toward alleviation (Figures 6O–S).

## Discussion

Antibiotic resistance is one of the major threats to global human health today (Ding et al., 2023; Leff et al., 2023). Since the 1960s, virtually no new classes of antibiotics have been discovered, and limited means for antibiotic development have led to a situation where some infectious diseases are gradually becoming untreatable (Wang et al., 2020). Therefore, there is an urgent need to identify an effective, residue-free alternative to antibiotics. Earthworms (referred to as “dì lóng” in traditional Chinese medicine) are regarded as natural medicines in traditional medicine, characterized by low toxicity and no significant residual toxicity. They contain multiple antimicrobial peptides with potent anti-pathogen activity, making them a potential carrier for developing novel antibiotic alternatives (Aira et al., 2024). This study systematically demonstrates for the first time that EH alleviates *E. coli* enteritis through multi-target synergistic mechanisms. These include direct antibacterial effects, inhibition of MAPK pathway-mediated inflammation, restoration of the intestinal physical barrier, and reshaping of the gut microbiome. This not only provides robust evidence for developing EH as an antibiotic alternative but also deepens our understanding of the complex pharmacological mechanisms of natural protein hydrolysates.

Notably, our *in vitro* antibacterial assay revealed that the crude EH exhibited significantly superior inhibitory effects (inhibition zone diameter of 12.24 mm) compared to the purified EAP (11.36 mm). This seemingly counterintuitive finding highlights the importance of synergistic interactions among multiple components within the crude hydrolysate. Unlike the purified EAP, which consists primarily of a defined set of antimicrobial peptides (> 98% purity), EH retains a complex repertoire of bioactive molecules, including various antimicrobial peptides, earthworm fibrinolytic enzymes, and other unidentified small peptides (Engelmann et al., 2005). We hypothesize that these diverse components act synergistically: antimicrobial peptides disrupt bacterial membrane integrity (Martins et al., 2024), while other constituents may simultaneously interfere with key bacterial metabolic pathways or enhance the membrane-penetrating efficiency of the antimicrobial peptides. In contrast, the highly purified EAP, despite its defined composition, lacks the “ensemble effect” conferred by the multi-component synergy present in the crude extract. This finding suggests that, when developing natural alternatives to antibiotics, retaining the complex multi-component matrix may offer therapeutic advantages over pursuing single highly purified compounds.

To establish a mouse model of *E. coli* enteritis, this study optimized the method described by Li et al. (2023), by inoculating mice with pathogenic *E. coli* at varying concentrations via intraperitoneal injection. Results showed that mice injected with a bacterial suspension at 1 × 10^7^ CFU/mL exhibited a 100% diarrhea rate and an 11.11% mortality rate. Necropsy revealed typical enteritis features including intestinal congestion and watery intestinal contents. Based on these clinical manifestations and pathological findings, the intraperitoneal injection of 1 × 10^7^ CFU/mL bacterial suspension was ultimately selected for subsequent therapeutic trials.

The minimum bactericidal concentration (MBC) is defined as the lowest concentration that kills ≥ 99.9% of bacteria and is considered the gold standard for evaluating bactericidal activity. Using the MBC as the *in vivo* dose ensures that the drug achieves effective bactericidal levels at the target site, thereby meeting the requirement for pathogen clearance in the treatment of infectious diseases. Following oral administration by gavage, drug concentrations at the target site may be reduced due to degradation in the gastrointestinal environment or incomplete absorption. Directly adopting the *in vitro* MBC as the *in vivo* dose compensates for such losses and ensures that effective bactericidal concentrations are reached locally in the intestine. Preliminary experiments conducted in our laboratory confirmed that the selected doses caused no observable toxicity in mice (e.g., no weight loss, abnormal behavior, or abnormal organ indices) and significantly alleviated diarrhea symptoms and intestinal pathological damage, indicating that these doses are both safe and effective. The positive control drug, gentamicin (GM), was also administered at its MBC (512 μg/mL) to ensure comparability among different interventions. Therefore, the MBC was selected as the dosing concentration for all three agents.

Following successful model establishment, the final body weight of mice in the model group was significantly lower than their initial weight at the trial’s start, whereas the blank control group exhibited normal weight gain. This aligns with Zhang’s findings,^1^ confirming that *E. coli* infection induces catabolism and weight loss. Additionally, the liver index in the model group significantly increased, suggesting potential inflammatory liver damage such as congestion and edema. Compared to the model group, treatment with EH, EAP, and GM all significantly reduced the liver index (*P* < 0.05), indicating that these three interventions effectively alleviated *E. coli*-induced liver injury.

Invasion by pathogenic *E. coli* disrupts the intestinal epithelial barrier, subsequently triggering inflammation and dysbiosis (Fang et al., 2023). This study observed consistent pathological changes, with the model group exhibiting structural disorganization, deepened crypts, disrupted villi, and lymphocytic infiltration in jejunal tissue. Calculating the ratio of villus length to crypt depth revealed a significantly lower ratio in the model group compared to other groups (*P* < 0.05). Treatment with EH, EAP, and GM all demonstrated reparative effects on the aforementioned intestinal damage, effectively improving the histopathological morphology of the jejunum.

Intestinal inflammation can be triggered by multiple factors, with pathogenic microbial infections being the most common cause. Bacterial infections disrupt the balance between pro-inflammatory and anti-inflammatory factors in the gut, typically leading to elevated levels of key pro-inflammatory mediators such as TNF-α and IL-6 (Roe, 2021). Based on this, this study established a mouse intestinal injury model through artificial infection with *E. coli* to investigate the anti-inflammatory mechanism of EH. Compared with the control group, the levels of TNF-α, IL-6, and IL-2, as well as their relative mRNA expression, were significantly elevated in the jejunal tissue of the model group (P < 0.05). This result not only confirmed the successful establishment of the intestinal inflammation model (Lu et al., 2020; Wang et al., 2018), but also revealed a complex inflammatory response: The surge in TNF-α and IL-6 signifies a robust pro-inflammatory state (Tanaka et al., 2014; Zelova and Hosek, 2013), whereas the elevation of IL-2, given its known function in suppressing inflammation by inducing regulatory T cells and the anti-inflammatory factor IL-10 (Zhou et al., 2019; Bando et al., 2020), may reflect the body’s self-regulatory mechanism activated to counterbalance excessive inflammation. In therapeutic interventions, EH, EAP, and GM all significantly suppressed the expression of these inflammatory factors. The combined efficacy indicates that EH can significantly mitigate *E. coli*-induced intestinal inflammatory damage by effectively downregulating the expression of core pro-inflammatory mediators such as TNF-α and IL-6, and potentially modulating IL-2-related immune balance pathways.

The MAPK signaling pathway (ERK, JNK, p38) serves as a key hub mediating intestinal inflammatory responses (Peng et al., 2025; Jiao et al., 2020). This study revealed that *E. coli* infection significantly activated this pathway (P-ERK, P-JNK, and P-P38 all increased, *P* < 0.05). Treatment with EH effectively reversed this process, significantly downregulating the phosphorylation levels of key proteins (*P* < 0.05). This indicates that the anti-inflammatory mechanism of EH is at least partially derived from its negative regulation of the MAPK signaling pathway. This inhibits the activation of downstream transcription factors such as NF-κB, thereby reducing the expression of pro-inflammatory factors like TNF-α and IL-6. This effect aligns with the reported mechanisms of certain natural hydrolysates (Lee et al., 2021; Cian et al., 2020). Although the present study cannot completely exclude the possibility of indirect effects, the following evidence suggests that EH may exert anti-inflammatory effects independently of its antibacterial activity. Specifically, the direct antibacterial activity of EH (MIC = 375 μg/mL) was weaker than that of the positive control GM (MIC = 256 μg/mL); nevertheless, the two agents exhibited comparable *in vivo* anti-inflammatory efficacy in terms of reducing the levels of TNF-α, IL-6, and IL-2, as well as suppressing MAPK phosphorylation. If MAPK inhibition were merely a secondary consequence of bacterial clearance, the agent with stronger antibacterial activity (GM) would be expected to produce superior anti-inflammatory effects, which is inconsistent with the observed results. Notably, EH is fundamentally a complex mixture dominated by antimicrobial peptides, retaining a variety of small peptides, enzymes, and other bioactive components, whereas the positive control EAP, which exhibited comparable *in vivo* efficacy, is a purified product of a single antimicrobial peptide. The similarity in their anti-inflammatory and barrier restorative effects further suggests that the multi-component synergy within EH, rather than the action of any single class of antimicrobial peptides, plays a critical role in its pharmacological activity. Furthermore, as a crude extract rich in multiple bioactive components, EH may contain peptides or small molecules capable of directly modulating immune signaling pathways. However, we acknowledge that the current study cannot fully exclude the contribution of indirect effects. Future research employing non-proliferative inflammatory models, such as LPS-induced inflammation, or using control agents with matched antibacterial activity, is warranted to further elucidate the direct regulatory role of EH on the MAPK pathway.

An intact intestinal barrier is crucial for maintaining gut homeostasis, with epithelial tight junctions (e.g., ZO-1, Occludin, Claudin-1) serving as the physical core preventing pathogen translocation (Pellegrini et al., 2023; Wells et al., 2022; Martínez-Augustin et al., 2025). This study demonstrates that *E. coli* infection markedly reduces the expression of these proteins, compromises barrier integrity, and triggers a robust inflammatory response, consistent with the reported vicious cycle mechanism of inflammation-barrier damage (Wang et al., 2023; Chen et al., 2024; Hou et al., 2022). Notably, treatment with EH, EAP, and GM effectively repaired the damaged intestinal barrier, manifested by a significant rebound in tight junction protein expression and a concurrent decrease in inflammatory factor levels. This effect aligns with findings from studies by Liu H. et al. (2023). and Chinese herbal maggot extracts (Tang et al., 2023), collectively revealing that enhancing barrier function by upregulating tight junction proteins to alleviate intestinal inflammation represents a viable therapeutic strategy.

Gut microbiota homeostasis is the cornerstone of intestinal health, and its disruption drives disease progression, making microbiota modulation an emerging therapeutic approach (Wan et al., 2023; Duan et al., 2024). Proteolytic products have been demonstrated to exert anti-inflammatory effects by selectively regulating the microbiota, such as enriching beneficial bacteria and suppressing pathogenic bacteria (Zhang et al., 2025; Sheng et al., 2022; Liu et al., 2021). This study confirms that *E. coli* infection induces typical dysbiosis, with significantly reduced abundances of both Firmicutes and Bacteroidetes. We further observed that the model group exhibited a decreased F/B ratio, while supplementation with EH significantly increased the F/B ratio. This finding aligns with the results reported by Li et al. (2025). This study is the first to explore the effect of EH supplementation on increasing the F/B ratio in the gut microbiota, providing a reference for subsequent research. Conversely, Proteobacteria abundance significantly increased. *E. coli* infection is characterized by Proteobacteria expansion (Bleich et al., 2023), and antibiotic use also elevates Proteobacteria proportions (Phyo et al., 2021). At the genus level, *E. coli* infection led to significant reductions in beneficial genera Bacteroides and Lactobacillus, while potentially pathogenic genera such as Helicobacter and Desulfovibrio significantly increased. This aligns with previous findings (Tsou et al., 2021; An et al., 2023). Diversity analyses—including Chao1, observed species, Shannon, Simpson indices, and abundance curves—demonstrated that EH significantly improved the reduced diversity and abundance caused by *E. coli* infection. Collectively, these findings suggest that selectively reshaping the gut microbiota and restoring microecological balance represent one of the key mechanisms by which EH differs from traditional antibiotics in treating *E. coli* enteritis.

Several limitations should be acknowledged in this study. A key methodological consideration of this study is the use of intraperitoneal (IP) injection, rather than the natural oral route, to establish an *Escherichia coli* enteritis model. While the oral gavage model more closely mimics the natural infection process, it presents significant technical challenges in mechanistic studies, including unstable colonization efficiency and the need for high bacterial doses or pretreatment to overcome gastric barriers. The IP injection model offers distinct advantages: (1) it ensures uniformity and reproducibility of the infectious dose, minimizing inter-individual variability; (2) it bypasses the complex gastric environment, allowing precise investigation of the host inflammatory response to a defined bacterial stimulus; and (3) it reliably induces severe and phenotypically consistent enteritis, providing a rigorous experimental platform for evaluating the anti-inflammatory and barrier-protective effects of earthworm hydrolysate. Furthermore, EH is a complex mixture, and the precise identity of all active components responsible for its multifaceted effects remains to be fully elucidated. Future studies should focus on isolating and characterizing the key bioactive molecules within EH and validating its efficacy in natural infection models across different animal species.

## Conclusion

In summary, this study clearly demonstrates that EH effectively treats *E. coli*-induced intestinal injury through a synergistic action of regulating inflammatory factors, inhibiting the MAPK pathway, upregulating tight junction protein expression in the gut, and modulating microbial balance. This achievement systematically elucidates its multifaceted therapeutic mechanisms, providing a robust theoretical foundation for developing EH as a promising antibiotic alternative drug while filling a research gap in its application for treating intestinal inflammation.

## Data Availability

The data presented in the study are deposited in the OMIX repository, accession number(s) OMIX016596.

## Supplementary material

Supplementary Figure 1 Graphical abstract. Blue arrows indicate the comparison between the *E. coli* group and the CON group; green arrows indicate the comparison between the EH group and the *E. coli* group.

Supplementary Figure 2 Immunofluorescence images showing the expression of p-ERK1/2, p-JNK1, and p-p38.
