# Supplementary figures and images for: EartEarthworm hydrolysate alleviates Escherichia coli-induced enteritis by suppressing MAPK pathway activation, enhancing intestinal barrier integrity, and modulating gut microbiota
Source: Front Microbiol. 2026 May 4;17:1810555. doi: 10.3389/fmicb.2026.1810555 (PMC13182235; doi:10.3389/fmicb.2026.1810555)

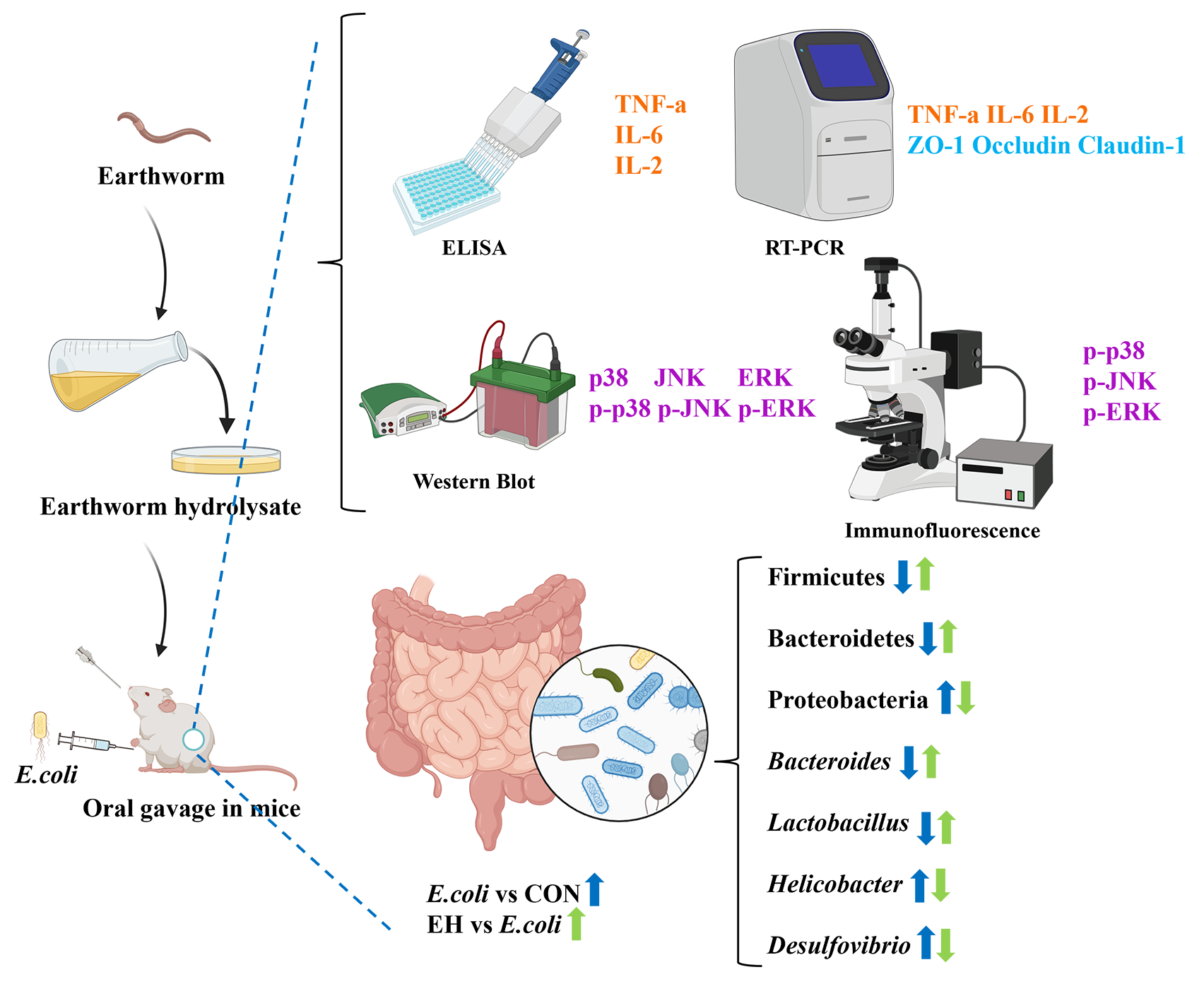

Supplement: Supplementary Figure 1 — Graphical abstract. Blue arrows indicate the comparison between the E. coli group and the CON group; green arrows indicate the comparison between the EH group and the E. coli group. [file Image_1.TIF]

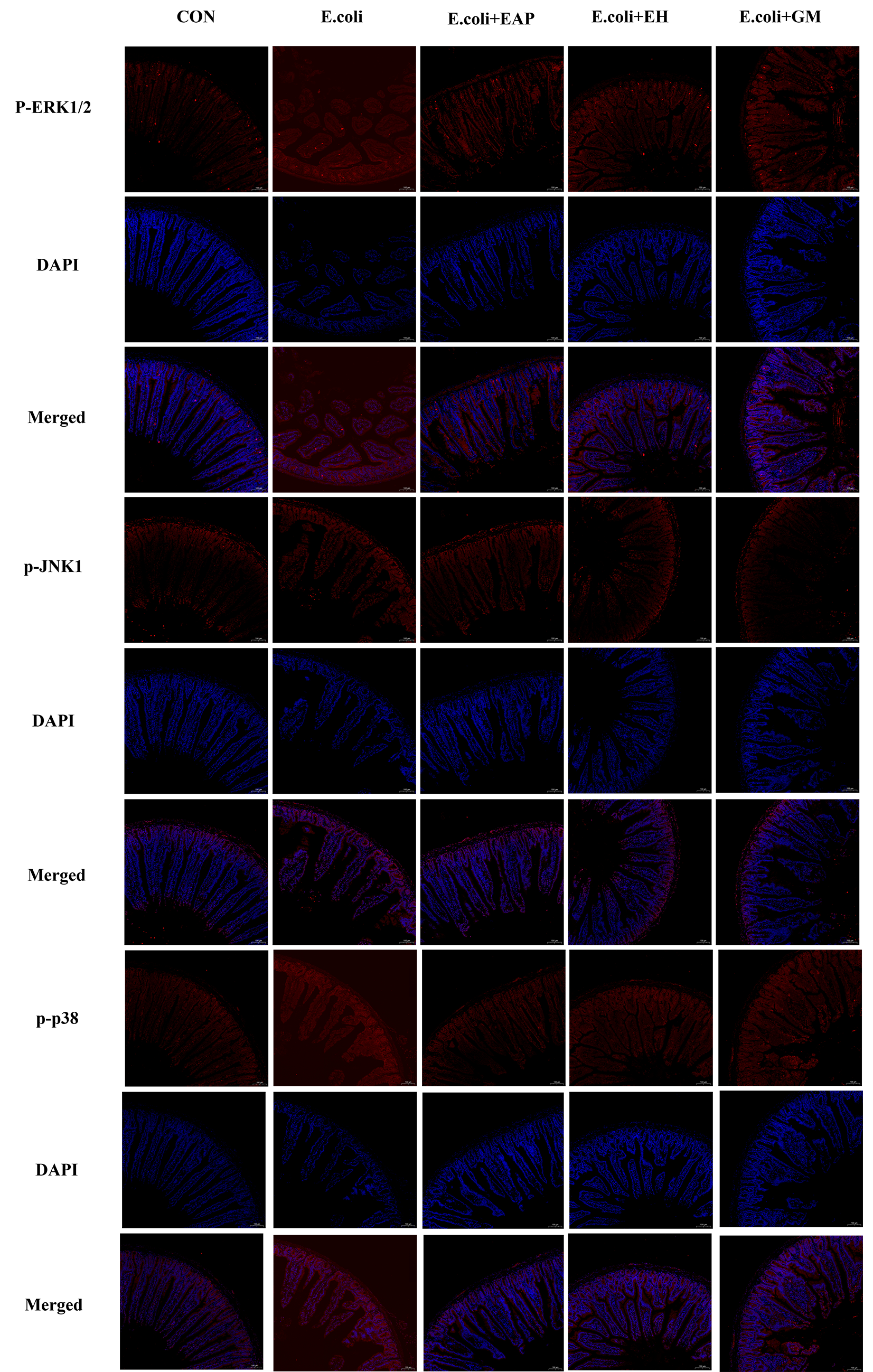

Supplement: Supplementary Figure 2 — Immunofluorescence images showing the expression of p-ERK1/2, p-JNK1, and p-p38. [file Image_2.TIF]
